# High-resolution 3D imaging of whole organ after clearing: taking a new look at the zebrafish testis

**DOI:** 10.1038/srep43012

**Published:** 2017-02-17

**Authors:** Maxence Frétaud, Laurie Rivière, Élodie De Job, Stéphanie Gay, Jean-Jacques Lareyre, Jean-Stéphane Joly, Pierre Affaticati, Violette Thermes

**Affiliations:** 1 INRA, UR1037 Fish Physiology and Genomics, F-35000 Rennes, France; 2 Tefor Core Facility, Paris-Saclay Institute of Neuroscience, CNRS, Université Paris-Saclay, 91190 Gif-sur-Yvette, France

## Abstract

Zebrafish testis has become a powerful model for reproductive biology of teleostean fishes and other vertebrates and encompasses multiple applications in applied and basic research. Many studies have focused on 2D images, which is time consuming and implies extrapolation of results. Three-dimensional imaging of whole organs recently became an important challenge to better understand their architecture and allow cell enumeration. Several protocols have thus been developed to enhance sample transparency, a limiting step for imaging large biological samples. However, none of these methods has been applied to the zebrafish testis. We tested five clearing protocols to determine if some of them could be applied with only small modifications to the testis. We compared clearing efficiency at both macroscopic and microscopic levels. CUBIC and PACT were suitable for an efficient transparency, an optimal optical penetration, the GFP fluorescence preservation and avoiding meaningful tissue deformation. Finally, we succeeded in whole testis 3D capture at a cellular resolution with both CUBIC and PACT, which will be valuable in a standard workflow to investigate the 3D architecture of the testis and its cellular content. This paves the way for further development of high content phenotyping studies in several fields including development, genetic or toxicology.

Testis has long been studied to better understand mechanisms of spermatogenesis, puberty or sterility disorders that are key issues in fundamental research, aquaculture and medical science. In the field of reproductive biology, the zebrafish has become a useful model, by means of many modern molecular tools (genome sequencing, fluorescent molecular staining, transgenic approaches). The zebrafish model also benefits of the recent advance in genome editing with the CRSIPR/Cas9 technology, which will eventually lead to the production of a high number of mutant lines to be analyzed^1^,^2^,^3^. Histological methods based on tissue sectioning are traditionally used to study gene expression patterns, tissue morphology or cellular content, in normal and experimental conditions. Apart from the laborious aspect of this approach, it implies extrapolation of results and provides only limited spatial information. A three-dimensional (3D) view of testis is now essential to better understand the testis 3D architecture and to allow accurate cellular enumeration.

3D-fluorescence imaging has made significant progress with the development of cutting-edge microscopy technologies. Nonetheless, imaging of large biological samples remains greatly hindered by the natural opacity of tissues. To overcome this limitation, several protocols have been developed that enhance tissue transparency by reducing light scattering, reviewed by Richardson and Lichtman^4^. All these clearing methods have been primarily developed on mouse neuronal tissue^5^,^6^,^7^,^8^,^9^. Whereas successful clearing and 3D imaging of other mouse organs or of other species have also been completed, attempts on zebrafish testis have not been reported to date. Here, we chose five of the available clearing techniques to be tested on adult zebrafish testis in order to determine if some of them could be applied with only small modifications. The five methods belong to each of the four main classes of clearing protocols including the high-refractive index aqueous solutions, the organic solvents, the hyperhydrating solutions and the tissue transformation methods^4^,^10^. Each of them has made a clear contribution. The two first methods belong to the first class and rely on the homogenization of refractive indices of medium and tissues by using a high-refractive index aqueous solution. The SeeDeepBrain (SeeDB) method^8^ uses a sugar-based solution and the Refractive Index Matching Solution (RIMS) is composed of the contrast agent iohexol^11^. SeeDB is a relatively rapid clearing method that preserves sample morphology and allows 2-photon imaging over several millimeters into the mouse brain. RIMS is a custom economical recipe that was originally developed as an alternative to the commercial FocusClear^11^. While the high-refractive index aqueous solution SeeDB was largely used alone for clearing biological samples, the RIMS has mainly been used with the PACT clearing method. The third method tested, named 3D imaging of solvent-cleared organs protocol (3DISCO), is the first organic solvents based protocol that allowed fluorescence preservation^12^. Dehydration and elimination of lipids by 3DISCO is very fast and leads to a high transparency of samples. The fourth method, named Clear Unobstructed Brain/Body Imaging Cocktails and Computational analysis (CUBIC), is based on an hyperhydration of samples by using aminoalcohols, urea and removal of lipids with detergent^9^. CUBIC combines a high clearing efficiency and a simple immersion protocol. Finally, PAssive CLARITY Technique (PACT) takes advantage of an hydrogel that stabilizes the tissue structure allowing removal of lipids by using detergent^11^. The use of passive removal of lipids and optimization of clearing reagents has proven to be effective for clearing without tissue deformation.

We tested the clearing efficiency of RIMS, SeeDB, 3DISCO, CUBIC and PACT methods on the adult zebrafish testis. We evaluated the sample transparency, the optical depth penetration and the tissue deformation. Nuclear staining with propidium iodide (PI) was chosen to test the imaging efficiency. Assessment of the preservation of the GFP fluorescence was performed by using a zebrafish transgenic line that express GFP in Sertoli cells^13^. Our study reports that CUBIC and PACT are suitable for an efficient transparency, an optimal optical penetration, the preservation the GFP fluorescence and avoiding meaningful tissue deformation. Finally, we successfully attempted to image whole cleared testis by using 2-photon microscopy after CUBIC or PACT clearing. Such a combination of tissue clearing and microscopy can be applied to investigate testis 3D architecture and cellular content. Furthermore, this paves the way for further development of high content phenotyping of abnormal zebrafish testes for diagnosis, drug discovery or high content mutant screening.

## Results

### Sample transparency

**Figure 1:**
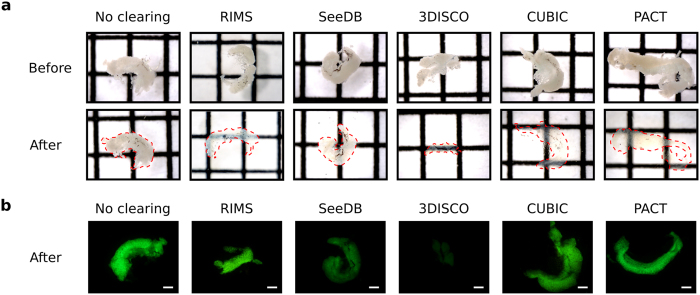
Comparison of the transparency and the GFP fluorescence of testes treated with different clearing methods. Testes were dissected from the zebrafish transgenic line *Tg*(*gsdf:GFP)* and cleared with RIMS, SeeDB, 3DISCO, CUBIC or PACT protocols. Testes were incubated in the refractive index matching solution of the last step of each protocol and imaged within 1 day. (**a**) Brightfield images of testes before and after clearing with the indicated methods. Transparency is assessed by the visualization of black lines situated underneath each sample. Dotted red line indicates the edge of testes after clearing. Square = 1.6 mm × 1.6 mm. (**b**) GFP fluorescence of cleared and non-cleared testes. The different clearing protocols used are indicated. Scale bar: 500 μm.

To assess the clearing efficiency of RIMS, SeeDB, 3DISCO, CUBIC and PACT protocols on zebrafish testes, samples were observed at a macroscopic level. The apparent transparency of all cleared samples was compared to that of non-cleared testes (*i*.*e*. testes incubated in PBS, Fig. 1a). Control samples displayed a whitish appearance and were completely opaque. RIMS treated samples notably displayed an improved transparency, whereas SeeDB had only a slight effect as compared with control. By contrast, CUBIC and PACT led to completely transparent testes. The most impressive result was obtained after 3DISCO clearing as 3DISCO-treated testes became almost invisible. These later also showed an important size decrease, as compared with other conditions.

### Fluorescence preservation

We tested whether RIMS, SeeDB, 3DISCO, CUBIC and PACT protocols were compatible with the observation of the endogenous GFP fluorescence. With this aim, we used a zebrafish transgenic line expressing the GFP in Sertoli cells^13^. In this transgenic line, the construct *Tg*(*gsdf:GFP)* encodes the GFP protein driven by the promoter of the zebrafish *GSDF* gene. Testes were cleared using the different methods and the fluorescence was observed afterward with a fluorescence macroscope (Fig. 1b). Although RIMS did not decrease fluorescence intensity, the SeeDB protocol greatly reduced the fluorescence intensity within the samples as compared with non-cleared control sample. The organic solvent-based method 3DISCO completely quenched the GFP fluorescence, even after being incubated only 15 minutes in dibenzyl ether (DBE, Supplementary Fig. S1). By contrast, testes treated with CUBIC and PACT protocols did not display any evident decrease of fluorescence intensity as compared with the control, indicating that both methods allowed preservation of fluorescence.

### Fluorescence recovery in depth

**Figure 2:**
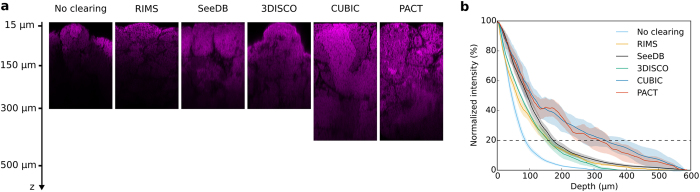
Comparison of the fluorescence recovery in depth from testes treated with different clearing protocols. Two-photon imaging of testes cleared with RIMS, SeeDB, 3DISCO, CUBIC or PACT protocols. (**a**) XZ planes of testes. Nuclei were stained with propidium iodide (in magenta). Laser intensity was set in order to be next to saturation at the beginning of the stack and no depth compensation was used. No brightness and contrast enhancement was applied. (**b**) Quantification of fluorescence intensity. Mean fluorescence intensity was normalized and plotted against the imaging depth. Mean ± SEM of 4–12 ROI acquired on 2–3 different testes.

Entire testes were stained with PI and sectioned with a vibratome to gain access to the nuclear staining in-depth. Imaging on transversal sections confirmed that deep nuclei were efficiently stained (Supplementary Fig. S2). We then assessed the optical penetration of 2-photon imaging after clearing with the different methods. Whole testes cleared and stained with PI were imaged by 2-photon microscopy, with no signal compensation in depth and no image post-processing (Fig. 2). XZ views of z-stacks revealed that the maximal signal recovery ranged in depth from 100 to 400 μm, depending on the clearing protocol (Fig. 2a). The mean fluorescence intensity was quantified through the z-stack and plotted as a function of depth (Fig. 2b). In the control condition, we observed an important decrease of the fluorescence intensity that reached 20% of the maximal intensity at 86 μm depth. By contrast, RIMS and SeeDB displayed a slower loss of fluorescence as compared with control condition. About 20% of signal intensity could indeed be recovered at 159 and 173 μm in depth respectively, indicating a substantial improvement of the optical penetration with these clearing protocols. For 3DISCO samples that displayed an important size reduction, the resulting curve was similar with a signal recovery of 20% at 157 μm. PACT and CUBIC were the most efficient protocols to recover fluorescence signal from deep regions, with 20% of the maximum intensity at 316 and 335 μm in depth, respectively.

### Depth-resolved imaging

**Figure 3:**
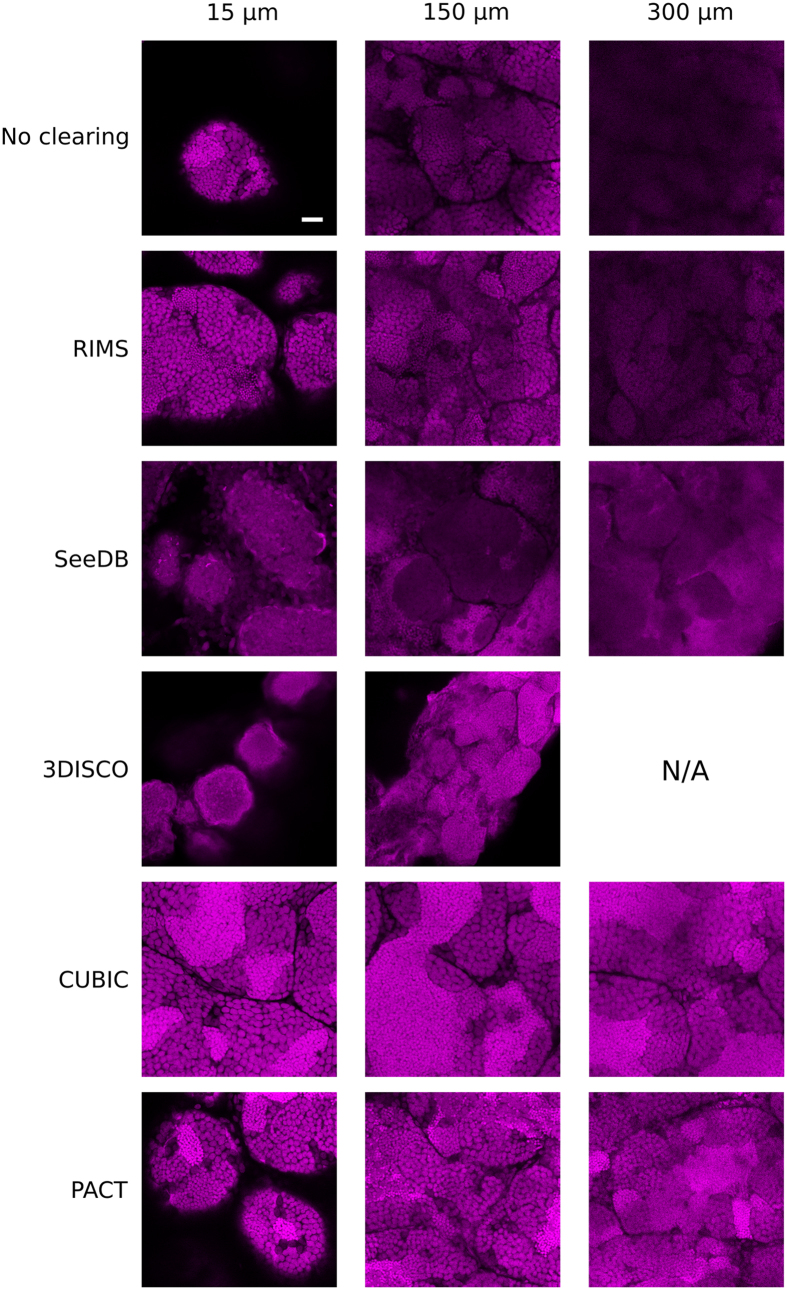
Comparison of the spatial resolution of images acquired from testes treated with different clearing protocols. Two-photon imaging of testes cleared with RIMS, SeeDB, 3DISCO, CUBIC or PACT protocols. XY planes of testes at three different imaging depths: 15 μm, 150 μm and 300 μm. Nuclei were stained with propidium iodide (in magenta). Laser intensity was set in order to be next to saturation at the beginning of the stack and no depth compensation was used. Brightness and contrast has been modified to assess spatial resolution. Images were acquired at a scanning speed of 400 Hz and at a resolution of 1024 × 1024 pixels with two lines average. N/A: Not available because there is no tissue at this depth. Scale bar: 50 μm.

For each cleared sample, we examined the spatial resolution of 2-photon microscopy images (Fig. 3). Given the z-limitation of imaging (described above), XY-planes of the different cleared-samples were compared at 15 μm, 150 μm and 300 μm. Images were acquired without laser compensation but image post-processing was applied (enhancement of brightness and contrast). No fluorescence was obtained for 3DISCO samples at 300 μm, due to the limited size of samples after treatment (see Fig. 1a). Different clusters of cells were easily distinguishable on optical sections. Spermatozoa nuclei were recognizable by their little size (about 5 μm) and their high fluorescence due to DNA compaction. These cells constitute large densely packed clusters of cells in the center of each seminiferous tubule. In control samples, nuclei were hardly resolved at 150 μm depth and almost no signal was recovered at 300 μm. Images obtained with SeeDB- and 3DISCO-treated samples displayed a poor resolution even at the beginning of the stacks. By contrast, nuclei were easily distinguishable with RIMS at 150 μm but a loss of resolution was observed at 300 μm. Finally, with CUBIC and PACT, the spatial resolution was sufficient to distinguish nuclei up to 300 μm.

### Comparison of 2-photon and confocal microscopy

**Figure 4:**
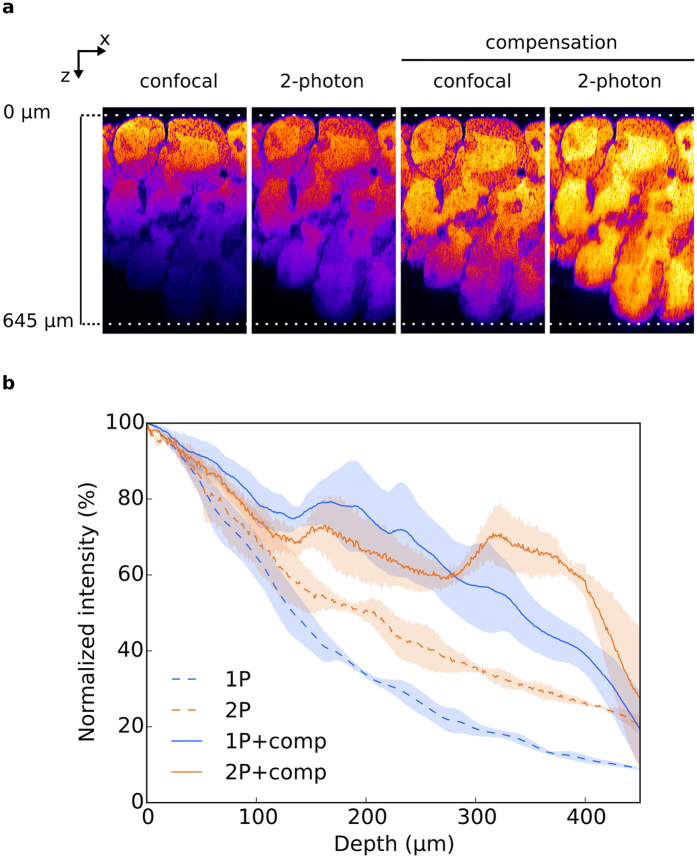
Comparison of confocal and 2-photon imaging on CUBIC cleared testes. (**a**) XZ planes of testis treated with CUBIC and acquired either by confocal or 2-photon microscopy, with or without laser compensation. No brightness and contrast enhancement was applied. Nuclei were stained with propidium iodide and pseudocolored. (**b**) Fluorescence intensity quantification. Mean fluorescence intensity was normalized and plotted against the imaging depth. All data are mean ± SEM of 3 ROI acquired on 1 testis.

We compared the 2-photon and 1-photon (confocal) imaging on CUBIC-cleared samples, with or without laser compensation. XZ orthoslices of the stacks revealed a deeper signal recovery with 2-photon microscopy without laser compensation, as compared with confocal microscopy. The imaging depth was similar with 2-photon and confocal microscopy with laser compensation (Fig. 4a). The mean fluorescence intensities were measured as a function of depth (Fig. 4b). When no depth compensation was applied, resulting curves indicated that the signal intensity of the 2-photon images decreases at a slower rate than that of confocal images. 50% of the signal was indeed recovered at 208 μm with the 2-photon, whereas the same percentage was recovered at 134 μm with the confocal. On the contrary, when applying depth compensation, we were able to improve the signal recovery with both confocal and 2-photon microscopy, and the whole thickness of the testis could be acquired with both imaging techniques.

### Nuclear size modification

**Figure 5:**
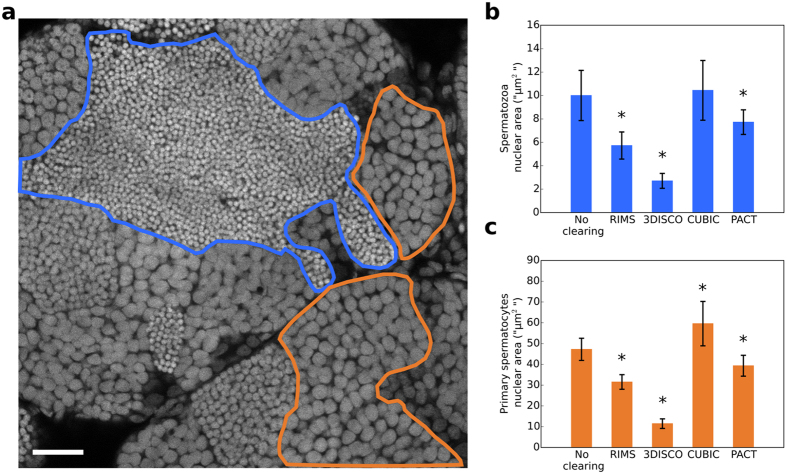
Comparison of the nuclear size of testes treated with different clearing protocols. (**a**) Optical section of a non-cleared testis. Nuclei were stained with propidium iodide. Nuclei of germ cells at different stages of differentiation are packed in distinct domains. A spermatozoa domain is delineated in blue and a primary spermatocyte domain is delineated in orange. For each clearing condition (RIMS, 3DISCO, CUBIC and PACT), the nuclear area of 55 spermatozoa and 55 primary spermatocytes was measured, spreading across 11 optical sections from two testes. (**b**) Nuclear area of spermatozoa. (**c**) Nuclear area of primary spermatocytes. All data are indicated as mean ± SD. *Indicates a significant difference as compared with PBS (p < 0.0001) using a one way ANOVA with Dunnet’s post-hoc test for multiple comparison. Scale bar: 25 μm.

In order to get insight into the effect of clearing on testis integrity, and based on the assumption that nuclear size modifications reflect cellular size changes, we measured nuclear sizes after clearing (Fig. 5). For each clearing condition, we measured the nuclear size of spermatozoa (Fig. 5a,b) and of primary spermatocytes (Fig. 5a,c), except for SeeDB since spatial resolution was too low. 3DISCO testes samples displayed an important decrease of spermatozoa and primary spermatocytes nuclear size, consistently with its global size decrease (Fig. 1a). With RIMS and PACT, samples also displayed a significant decrease of the nuclear size of both cell types. By contrast, CUBIC treatment led to no nuclear size modification of spermatozoa nuclei and a slight increase of the primary spermatocytes nuclear size (Fig. 5c).

### Whole testis 3D imaging after CUBIC-clearing

**Figure 6:**
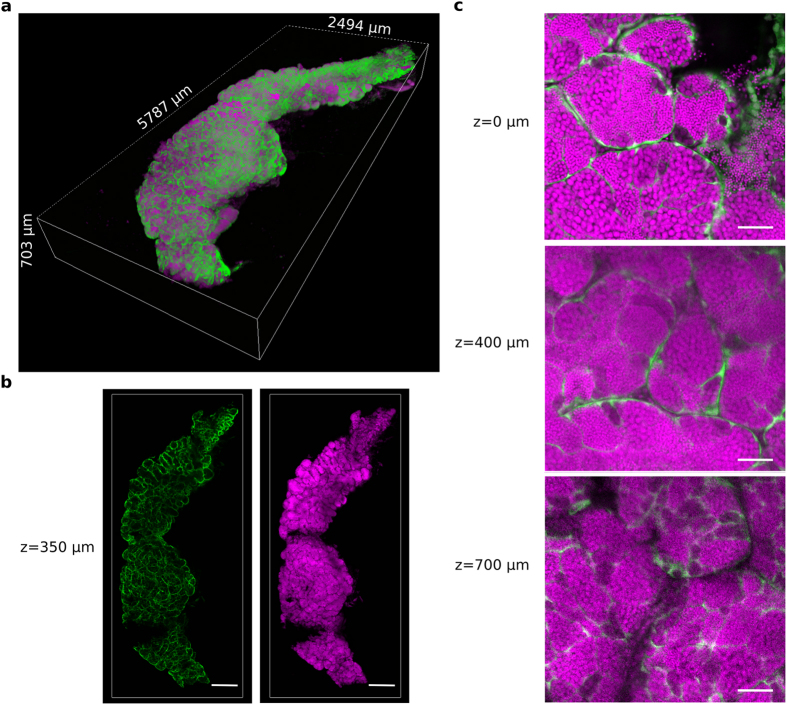
A 3D reconstruction of a whole zebrafish testis cleared with CUBIC. A testis was dissected from the zebrafish transgenic line *Tg*(*gsdf:GFP)* and cleared with the CUBIC protocol. All nuclei are in magenta (propidum iodide). The somatic Sertoli cells are in green (endogenous *GFP* fluorescence). (**a**) 3D rendering of the whole CUBIC-cleared testis. (**b**) 2D optical sections of the testis at 350 μm in depth. (**c**) Magnified view of 2D optical sections at 0 μm, 400 μm and 700 μm in depth. Images were acquired in 8 bits at a scanning speed of 600 Hz and at a resolution of 512 × 512 pixels with two lines average. Scale bars: 500 μm (**b**) and 50 μm (**c**).

A Testis collected from the transgenic line *Tg*(*gsdf:GFP)* was cleared following the CUBIC method and imaged by 2-photon microscopy. A total volume of 5.787 mm × 2.494 mm × 0.703 mm was acquired (Fig. 6a and Supplementary Video S3,S4). Imaging of the whole testis took about 30 hours in our conditions (voxel size: 0.577 μm × 0.577 μm × 1 μm) and it generated 57 GB of data. Images were acquired with laser compensation and contrast enhancement was applied. Nuclear staining appeared homogeneous at different depths (Fig. 6b). Although sperm nuclei are highly densely packed, the resolution was still sufficient at 700 μm deep, allowing discriminating nuclei (Fig. 6c). Acquisition of GFP fluorescence until 700 μm indicated that the CUBIC clearing method allow recovery of GFP signal through the whole organ. Sertoli cells were present in all regions of the testis, lining the tubular walls and spermatocysts. Similar result was obtained with a testis cleared with PACT and imaged by 2-photon microscopy (Supplementary Figure S5 and Supplementary Videos S6 and S7).

### 3D PACT-imaging of germinal niches

**Figure 7:**
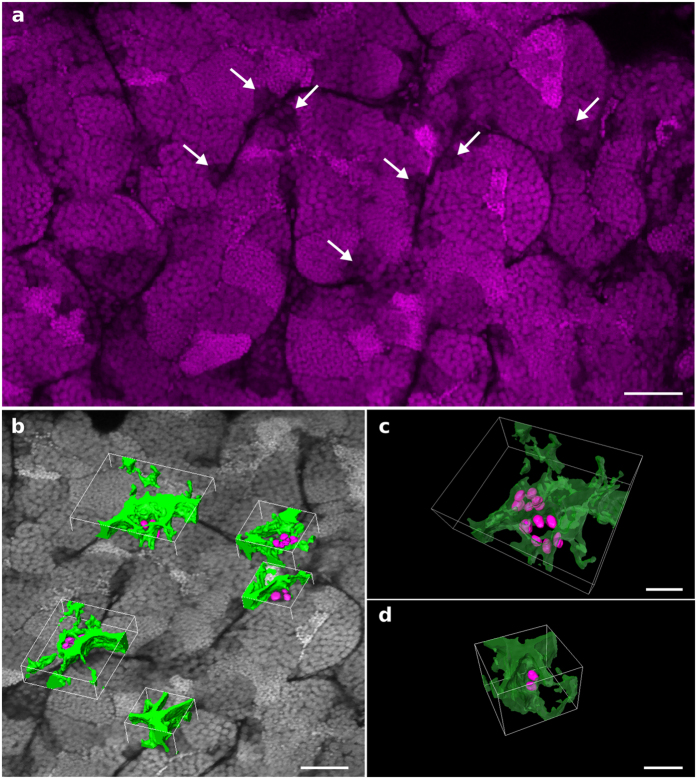
PACT 3D imaging of a zebrafish testis to study the architecture of germinal niches. The testis dissected from a *Tg*(*gsdf:GFP)* transgenic line was stained with propidium iodide and cleared with the PACT protocol. Imaging and 3D reconstruction of the whole testis were performed as shown in Supplementary Fig. S5 and Supplementary Videos S6 and S7. (**a**) A 2D optical section at 237 μm in depth. Nuclei of undifferentiated spermatogonia are identified through the testis by their larger volume and low fluorescence intensity (arrows). (**b**) Examples of 3D surface reconstructions of germinal niches containing clustered undifferentiated spermatogonia. (**c**) High magnification view of two nearby niches located in adjacent seminiferous tubules and displaying six nuclei each. (**d**) High magnification view of a niche displaying two nuclei of undifferentiated spermatogonia. Nuclei are in grayscale or magenta. Surface reconstructed Sertoli cells and nuclei of undifferentiated spermatogonia are in green and magenta, respectively. Scale bars: 50 μm (**a**,**b**) and 20 μm (**c**).

The 3D organization of the male germinal niches is poorly documented. Here, we analyzed the composition of germinal niches containing undifferentiated A spermatogonia, by using the 3D reconstruction data of PACT-cleared testis (see Supplementary Figure S5 and Supplementary Videos S6 and S7). Undifferentiated A spermatogonia include spermatogonial stem cells that are recognizable by the larger volume of their rounded nucleus, a poorly fragmented central nucleolus and the lower condensation of their chromatin, which results in a lower fluorescence intensity (Fig. 7a, arrows). The germinal nuclei were segmented from the 3D reconstruction data (Fig. 7b). Resulting 3D surface reconstructions display the number of undifferentiated spermatogonia in the germinal niches and their relative position regarding the surrounding Sertoli cells (Fig. 7c,d and Supplementary Videos S8 and S9). Our observations revealed that most of the germinal niches contained 2, 4 or 6 undifferentiated spermatogonia, which formed twisted chains remaining at the periphery of the seminiferous tubules rather than a cystic organization (Fig. 7c).

## Discussion

While recent advances in tissue clearing now allow successful 3D imaging of various mouse organs, this methodology was so far not available for zebrafish testis. Here, we tested five of the main tissue clearing methods available to determine if some of them could be applied with only minor changes. Of the different protocols tested, CUBIC and PACT were the only two methods allowing imaging of the whole zebrafish testis at a cellular resolution. Using PACT clearing and 3D imaging, we were then able to investigate the cellular organization of clustered undifferentiated spermatogonia in the germinal niches. We noted that most of undifferentiated spermatogonia were not isolated, as previously proposed^14^, but rather regrouped in chains of 2, 4 or 6 cells at the periphery of the seminiferous tubules.

To our knowledge, this is the first study that compares clearing efficiency on testis. Although mouse testis has been already cleared by BABB^15^, CLARITY^16^ and its recent variant ACT-PRESTO (Active Clarity Technique-Pressure Related Efficient and Stable Transfer of macromolecules into Organs)^17^, no detailed performance evaluation on testis has been reported before. We analyzed efficiency of five clearing methods at macroscopic and microscopic levels. Results are summarized in Table 1. We observed that 3DISCO, CUBIC and PACT rendered testes completely transparent. RIMS, CUBIC and PACT allowed preserving the GFP fluorescence better than 3DISCO and SeeDB. All methods induced size changes that were minimal with CUBIC and PACT. The best resolved imaging in depth was obtained with CUBIC and PACT that reached several hundreds of μm under the surface, thus allowing whole organ imaging. Overall, CUBIC and PACT are thus the two most suitable methods for clearing and imaging zebrafish testis. The efficiency of clearing by CUBIC and PACT could now be tested on testis of other species, including mammals. However, this does not exclude in the next future the possibility of improving or developing any other protocols presented in the literature.

The native GFP fluorescence of the transgenic line *Tg*(*gsdf:GFP)* was importantly reduced in testis with 3DISCO and SeeDB, although it was properly preserved with RIMS, CUBIC and PACT. Given the chemical treatments that were applied to testis samples, it is not surprising that the fluorescence was somewhat impaired, if not totally suppressed. The fluorescence of GFP and variants not only depends on the pH and the temperature, but also on the protein secondary structure^18^. Detergents, organic solvents and dehydration processes of some clarifying protocols may lead to an important decay of the GFP fluorescence, likely through denaturation of the GFP proteins. Generally, the use of 3DISCO, an organic-solvent based protocol, leads to an important decay of the GFP fluorescence^19^,^20^. Hence, we were very careful in imaging testis samples the first day following the end of the 3DISCO clearing protocol, as recommended by Erturk *et al*.^6^. In spite of this precaution, we almost totally lost the fluorescence of our GFP transgenic line that was already quenched after being incubated 15 minutes in DBE (Supplementary Fig. S1). It is worth noting that most clearing methods are demonstrated with *thy1* mouse transgenic lines that are well known for their strong fluorescence, as reviewed by Silvestri *et al*.^10^. It is thus most likely that the GFP fluorescent signal of our transgenic line was not strong enough to withstand the protocol stringency. The use of another transgene, as done in the present study, is useful for a more realistic evaluation of the fluorescence preservation. However, some efforts have recently been made to adjust organic-solvent based protocols in order to limit the fluorescence decay. The fluoclearBABB method recommends the use of either the 1-propanol or the *tert*-butanol (adjusted to a basic pH) during the dehydration step^21^. The uDISCO (ultimate DISCO) method recommends the use of the *tert*-butanol and a diphenylether-BABB mix instead of the dibenzylether used in the original 3DISCO^22^. In these studies, authors observe preservation of the fluorescent proteins over months after clearing. Regarding SeeDB, it was described as a method that preserves EYFP fluorescence, however another study reported a decrease of the GFP fluorescence, consistently with our results^8^,^23^. This might be explained by the dehydration of cells due to the use of highly concentrated fructose solutions^18^. Interestingly, a more recent study published a new efficient high-refractive index solution SeeDB2 that has a better clearing performance and fluorescence preservation than SeeDB^24^. Whereas SeeDB is a fructose-based solution, SeeDB2 is composed of the contrast agent iohexol, similarly to RIMS. Authors show that the combination of iohexol with various detergents can efficiently facilitate optical clearing and preserve fluorescence without introducing any morphological damage. Finally, and consistently with our results, the CUBIC and PACT methods that can be expected to quench the fluorescence by the use of detergents, usually allow its preservation^11^,^25^,^26^. Overall, our data and those of the literature prove that the preservation of the GFP signal is not always guaranteed. The use of transgenic lines that strongly express GFP is recommended to ensure sufficient fluorescence intensity for imaging.

In most cases, the size of tissues is used to evaluate deformations after clearing, as it was successfully done for whole mouse brain or mouse brain slices^16^,^17^. However, the testis is not a structured and oriented tissue in contrast to the brain. The shape of testis varies according to individuals, preventing direct comparison. Based on the assumption that nuclear size modifications may reflect cell volume variation, we analyzed the size of nuclei in testes after treatment with the different clearing methods. Surprisingly, we were not able to measure the nuclear size in SeeDB treated samples since spatial resolution of pictures was too low. 3DISCO-testes showed an important global size reduction (Fig. 1) and a significant nuclear size decrease (Fig. 5), which supports our assumption. This result is also consistent with previous data. 3DISCO have been reported to induce important shrinkage of the brain^6^. Conversely, we observed a slight but significant increase of nuclear volumes with CUBIC. Urea-based clearing methods such as CUBIC have already been shown to promote a volume expansion due to tissue hyperhydration^4^,^9^,^19^. However, the original study showed a return to the initial size after incubation in CUBIC2^9^. The size fluctuation of cleared tissues anyhow depends on water fluxes between inside and outside of cell that regulate the osmotic pressure. Authors of the initial CUBIC protocol recently proposed to add a supplementary step of immersion in a ½-CUBIC2/PBS solution in order to minimize sample dilatation^27^. In our study, PACT also slightly reduced the nuclear size of samples, similarly to RIMS. It has been shown that PACT and CLARITY-derived methods induce a volume expansion during the step of lipid removing, and that immersion in the RI matching solution allows samples to return to the initial volume^7^,^11^,^28^. Fine changes of osmolality of RIMS solution in the final step of the PACT protocol should lead to cleared samples with limited size modification. Taken together, our data show that PACT is a suitable method that avoids tissue deformation.

Although the macroscopic clearing efficiency was the most powerful with 3DISCO in our hands, this solvent-based protocol is based on the use of toxic chemical reagents and results in very hard samples that tend to break, thus complicating the sample manipulation. In addition, we were not able to get resolved images by 2-photon microscopy, although the strong sample shrinkage should have made them easier to acquire in terms of time and data volume. One of the main advantages of CUBIC over PACT is its easiness of achievement. Other studies clearly indicate that CUBIC is an efficient clearing method for various mouse organs^20^,^27^,^29^. Nevertheless, the time requirement for the whole protocol is quite long. Further studies will evaluate protocols with shorter incubation in each CUBIC solution. We found that the RI matching solution CUBIC2 was very viscous and was not well adapted for mounting due to bubble formation. In a newer version of CUBIC protocol, authors recommend to immerse samples in a low-viscosity immersion oil mix after CUBIC2 incubation^27^. Moreover conservation in CUBIC2 was not advised, since evaporation made solution still harder as precipitation may occur. We anticipate that this solution could be replaced with another refractive index matching solution previously described like RIMS, sRIMS or 2,2′-thiodiethanol (TDE)^11^,^30^,^31^. Alternatively, samples could be imaged directly in CUBIC1^9^,^20^.

Using CUBIC and PACT, and optimal imaging conditions, we successfully attempted to image whole testis thickness (*i*.*e*. 700 μm) at a cellular resolution by using 2-photon microscopy, which shows the great efficiency of these methods on the zebrafish testis. It is well demonstrated, mainly on brain, that PACT-clearing and imaging with 2-photon/confocal microscopes allows imaging with a high resolution in depth up to more than 1 mm^7^,^9^. Here, it was not possible to test the resolution beyond 700 μm, given the limited size of zebrafish testes.

Overall, our work allowed for the first time the 3D imaging of whole zebrafish testis. The comparison of several clearing protocols showed that CUBIC and PACT are the most appropriate among the large number of protocols available in the literature. In combination with 2-photon microscopy, they can be applied to investigate testis 3D architecture and cellular content. Application of these protocols on testis of other species, including mammal, will be of utmost interest. This study is also the first step toward the development of high content phenotyping workflows of abnormal zebrafish testes for diagnosis, drug discovery or mutant screening.

## Methods

### Ethics statement

All experimental procedures used in this study followed the recommendations of the French and European Union regulation on animal welfare and were approved by the INRA LPGP-Animal Care and Use Committee (N°Z-2015-30-VT and Z-2015-127-VT-MF).

### Zebrafish breeding and collection of testes

Adults of the *Tg*(*gsdf:GFP)* zebrafish line were raised at 28 °C until adulthood (aged from 4 to 5 months) in the INRA-LPGP fish facility^13^. For testes dissection, adult male zebrafish were euthanized by immersion in a lethal dose of 2-phenoxyethanol (0,8 mg/ml). Testes were fixed overnight at 4 °C by 4% paraformaldehyde (PFA) in 0,01 M phosphate buffer saline pH 7.4 (PBS). After washing in PBS, testes were conserved in PBS +0,05% (w/v) sodium azide (S2002, Sigma-Aldrich) at 4 °C.

### Clearing protocols

#### RIMS

6.66 g of Histodenz^TM^ (D2158, Sigma-Aldrich) was dissolved in 5 ml of PBS 0,02 M +0,05% sodium azide^11^. Samples were incubated in RIMS during 24 h at room temperature.

#### SeeDB

SeeDB protocol was conducted as described by Ke and collaborators^8^, with modifications. Testes were immersed successively in 20% (w/v) D-(-)-fructose solution (F0127, Sigma-Aldrich) during 1 h, 40% (w/v) fructose during 1 h, 70% (w/v) fructose during 2 h 30 minutes, 100% (w/v) fructose during 4 h and 80,2% (w/w) fructose during 16 h. All steps were performed at room temperature on a rotating wheel. All fructose solutions were prepared in distilled water and contained 0,5% α-thioglycerol (M1753, Sigma-Aldrich).

#### 3DISCO

Samples were cleared by a simplified 3DISCO protocol^32^. All steps were performed at room temperature on a rotating wheel. Testes were incubated successively in 50% (v/v) tetrahydrofuran (THF) (186562, Sigma-Aldrich) during 30 minutes, 80% (v/v) THF during 30 minutes and 100% (v/v) THF during 30 minutes twice. Testes were incubated in dichloromethane (270997, Sigma-Aldrich) during 15 minutes. Samples were then immersed in dibenzyl ether (DBE, 108014, Sigma-Aldrich) at least 15 minutes.

#### CUBIC

Protocol was conducted as described previously^9^. Testes were first incubated during 3 days at 37 °C in CUBIC-1 reagent composed of 25% (w/w) urea (GE17-1319-01, Sigma-Aldrich), 25% (w/w) N,N,N′,N′-Tetrakis(2-Hydroxypropyl)ethylenediamine (122262, Sigma-Aldrich) and 15% (w/w) triton X-100 (X-100, Sigma-Aldrich) in distilled water. The CUBIC-1 reagent was renewed once the second day. Samples were washed in PBS several times at room temperature. Samples were then immersed overnight at room temperature in CUBIC-2 reagent composed of 50% (w/w) sucrose (S-9378, Sigma-Aldrich), 25% (w/w) urea, 10% (w/w) triethanolamine (90279, Sigma-Aldrich) and 0,1% (v/v) triton X-100 in distilled water. CUBIC-2 solution was renewed and samples were incubated 1 day at 37 °C. Samples were agitated on a rotating wheel during the whole protocol.

#### PACT

Fixed testes were subjected to clearing following CLARITY protocol^7^,^11^ with some tissue-specific adaptations: samples were infused in a pre-cooled (4 °C) solution of freshly prepared hydrogel monomers (0.01 M PBS, 0.25% (w/v) VA-044 initiator, 5% (v/v) DMSO, 1% (w/v) PFA, 4% (w/v) acrylamide and 0.0025% (w/v) bis-acrylamide) for 2 days at 4 °C. After degassing the samples the hydrogel polymerization was triggered by replacing atmospheric oxygen with nitrogen in a desiccation chamber for 3 h at 37 °C. Samples were cleaned from superfluous hydrogel and transferred into embedding cassettes for lipid clearing. Passive lipid clearing was performed at 40 °C for 8 days in the clearing solution (8% SDS (w/v), 0.2 M boric acid, pH adjusted to 8.5) under gentle agitation. Subsequently the samples were thoroughly washed in PBS +0.1% (v/v) tween at room temperature with gentle agitation for 2 days. Samples were then immersed in RIMS during 24 h at room temperature.

### Nuclear staining

Nuclear staining was performed before clearing for RIMS, SeeDB and 3DISCO, and after clearing for CUBIC and PACT. With these latter, the nuclear staining was performed before incubation in the refractive index solution (*i*.*e*. in CUBIC2 and RIMS respectively). Whole testes were incubated in PI (P4864, Sigma-Aldrich) diluted at 10 μg/ml in PBS +0.1% (v/v) tween +0,05% (w/v) sodium azide at room temperature for 2 days. Samples were then washed overnight in PBS +0.1% (v/v) tween +0,05% (w/v) sodium azide at room temperature.

### Macroscopic imaging

Reflected light pictures were acquired using a Zeiss stereomicroscope with an Axiocam digital camera. Fluorescence pictures were taken using Nikon AZ100 macroscope equipped with a DS-Ri1 digital camera.

### 3D fluorescence imaging

Mounting and imaging were performed at the end of the clearing protocol for all conditions. Samples were mounted under #0 coverslips (Secure seal, ems) with 5 spacers of 120 μm each in their respective final clearing solution. To assess the deepness of the PI staining, testes were embedded in 4% low gelling temperature agarose (A9414.Sigma-Aldrich) and examined using the VibMic combination system (Leica^®^). The VibMic enables accessibility of deep structures of large whole-mount specimens by the combination of a vibratome (VT1200S) with a SP8 confocal microscope allowing successive mechanic sectioning and block-face imaging. Imaging was performed with a Leica SP8 two-photon/confocal hybrid microscope with a 25X/0,95NA HCX IRAPO water immersion objective (11506323, Leica microsystems). For confocal microscopy, GFP and PI were excited by 488 nm and 552 nm lasers respectively and fluorescence was detected by PMT. For 2-photon microscopy simultaneous excitation of PI and GFP was performed at 850 nm with a Chameleon Vision II laser (Coherent) and fluorescence was detected by NDD HyD detectors (Leica microsystems) with BP 525/50 and BP585/40 filters respectively.

### Data analysis

Enhancement of brightness and contrast, scaling and measurements were done with Fiji^33^. For quantification of fluorescence decrease (Figs 2 and 4), a ROI of 300 × 300 pixels was set on the PI channel. The mean intensity inside the ROI was calculated for each slice of the stack. The highest mean intensity was set as the first point and normalized to 100%. Brightness and contrast has been modified in Fig. 3 as follows: set display range PBS 150 μm: [0–100]; PBS 300 μm: [0–20]; RIMS 150 μm: [0–100]; RIMS 300 μm: [0–60]; SeeDB 150 μm: [0–160]; SeeDB 300 μm: [0–40]; 3DISCO 150 μm: [0–120]; 3DISCO 300 μm: [0–25]; CUBIC 150 μm: [0–150]; CUBIC 300 μm: [0–80]; PACT 150 μm: [0–150]; PACT 300 μm: [0–80]). For nuclear size assessment (Fig. 5), the area of 55 sperm nuclei and 55 spermatocyte nuclei were measured manually with the freehand selection tool. To minimize the data volume, images of the whole CUBIC-treated testis were scaled to 0.7 in x and y. Data were opened in Amira 6.0.1 using the XLVolume extension on 64-bit Windows 7 computer equipped with an Intel Xeon E-1620 3.6 GHz, a Quadro K600 graphic card and 32 Gb of RAM. 3D reconstruction was performed using volume-rendering object with maximum intensity projection option. Sertoli cells were segmented by threshold adjustment and spermatogonial stem cells nuclei were segmented manually. A 3D surface rendering was obtained using surface view object. Statistical analysis was performed with the Prism 7 trial software.

## Additional Information

**How to cite this article**: Frétaud, M. *et al*. High-resolution 3D imaging of whole organ after clearing: taking a new look at the zebrafish testis. *Sci. Rep.* **7**, 43012; doi: 10.1038/srep43012 (2017).

**Publisher's note:** Springer Nature remains neutral with regard to jurisdictional claims in published maps and institutional affiliations.

## Supplementary Material

Supplementary Information

Supplementary Video S3

Supplementary Video S4

Supplementary Video S6

Supplementary Video S7

Supplementary Video S8

Supplementary Video S9

## Figures and Tables

**Table 1:** Comparison of clearing techniques tested in this study.

	Time	Ease of protocol	Clearing efficiency	GFP fluorescence preservation	2-photon optical penetration	Nuclear size alteration
RIMS	1 day	Easy	Medium	yes	+	Decrease
SeeDB	1 day	Easy	Weak	no	+	Not measured
3DISCO	<1 day	Easy but manipulation of toxic compounds	Strong	no	+	High decrease
PACT	13 days	Difficult	Strong	yes	+++	Decrease
CUBIC	5 days	Easy	Strong	yes	+++	Increase
