# Supplementary material for: High-resolution 3D imaging of whole organ after clearing: taking a new look at the zebrafish testis
Source: Sci Rep. 2017 Feb 17;7:43012. doi: 10.1038/srep43012 (PMC5314416; doi:10.1038/srep43012)
Supplement: Supplementary Information [file srep43012-s1.pdf]

Whole three-dimensional imaging after tissue clearing: taking a new look at the zebrafish testis

Maxence Frétaud[1], Laurie Rivière[2], Élodie De Job[2], Stéphanie Gay[1], Jean-Jacques Lareyre[1], Jean-Stéphane Joly[2], Pierre Affaticati[2], Violette Thermes[1*].

[1]INRA, UR1037 Fish Physiology and Genomics, F-35000 Rennes, France

[2]Tefor Core Facility, Paris-Saclay Institute of Neuroscience, CNRS, Université Paris-Saclay, 91190 Gif-sur-Yvette, France

Corresponding author: Violette Thermes, violette.thermes@inra.fr

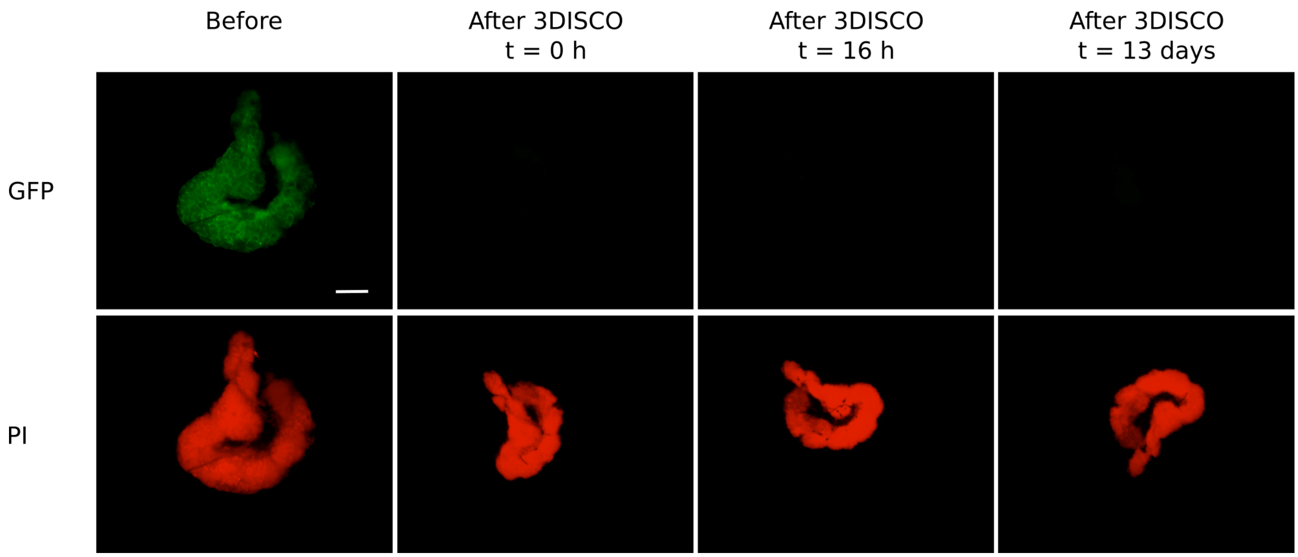

Supplementary Fig. S1: Fluorescence preservation within testis after 3DISCO clearing.

The testis was dissected from the zebrafish transgenic line *Tg(gsdf:GFP)*, stained with propidium iodide and cleared by using 3DISCO. Fluorescence of GFP and propidium iodide were both acquired before and after clearing at different time points. t = 0 h corresponds to the end of the last step of the protocol (15 minutes in DBE). Scale bar : 500 µm.

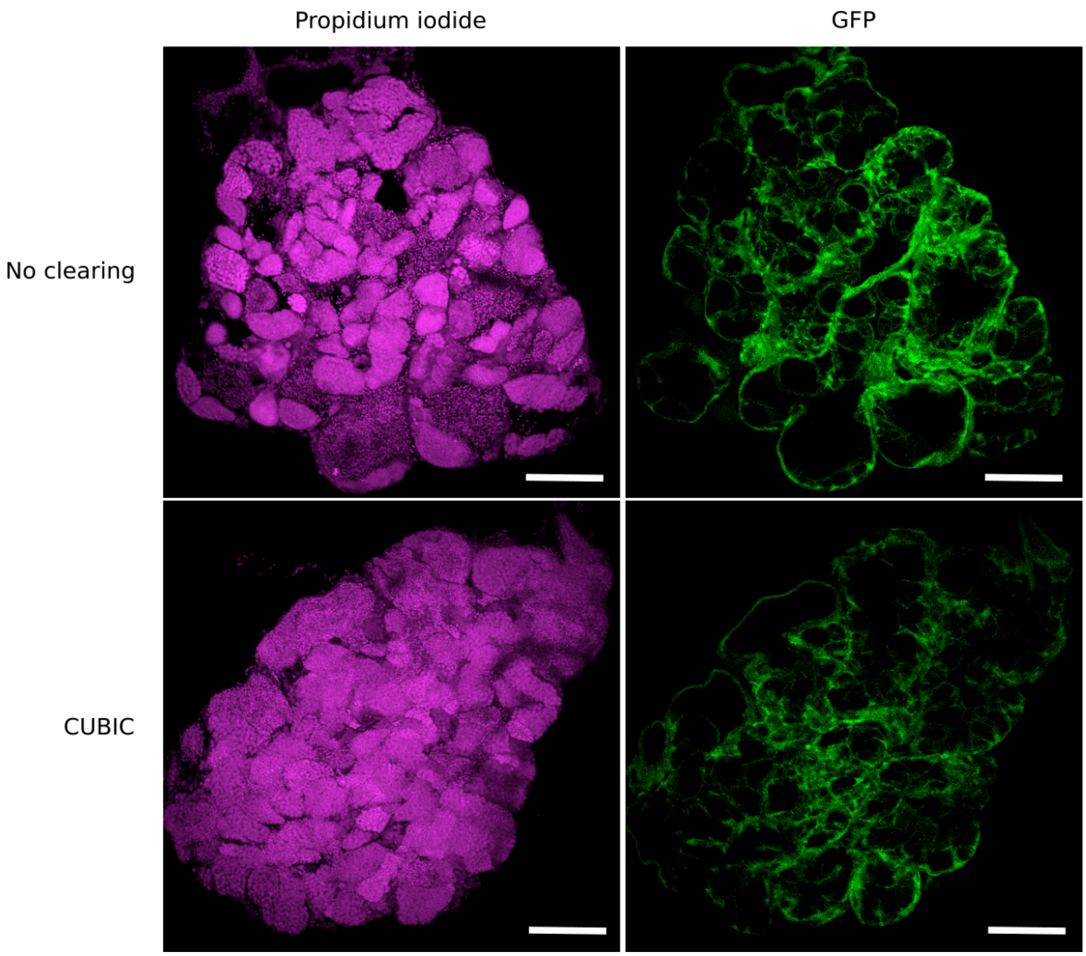

Supplementary Fig. S2: Confocal imaging of transverse vibratome sections of CUBIC-cleared and uncleared testes.

Testes were dissected from the zebrafish transgenic line Tg(gsdf:GFP), cleared or not with CUBIC and stained with propidium iodide. Transverse sections were performed with a vibratome to assess the nuclear staining in depth. Confocal images show that all nuclei in the center of the sample are efficiently stained. Images correspond to maximal projections of 10 optical slices (z-step = 3 µm). Nuclei are in magenta and Sertoli GFP cells in green. Scale bar: 100 µm.

Supplementary Video S3: 3D reconstruction of a whole testis cleared by the CUBIC method.

The movie shows the 3D rendering of a whole testis dissected from the zebrafish transgenic line *Tg(gsdf:GFP)*. Nuclei are in magenta and Sertoli GFP cells in green. Volume size: 5.787 mm x 2.494 mm x 0.703 mm.

Supplementary Video S4: z-stacks of the whole testis cleared by the CUBIC method.

The movie shows the 3D rendering of a whole testis dissected from the zebrafish transgenic line *Tg(gsdf:GFP)*. Sertoli GFP cells are in green. A series of 2D-optical slices showing GFP in Sertoli cells (z-step: 1μm). A series of 2D optical slices showing nuclear staining by propidium iodide (z-step: 1μm).

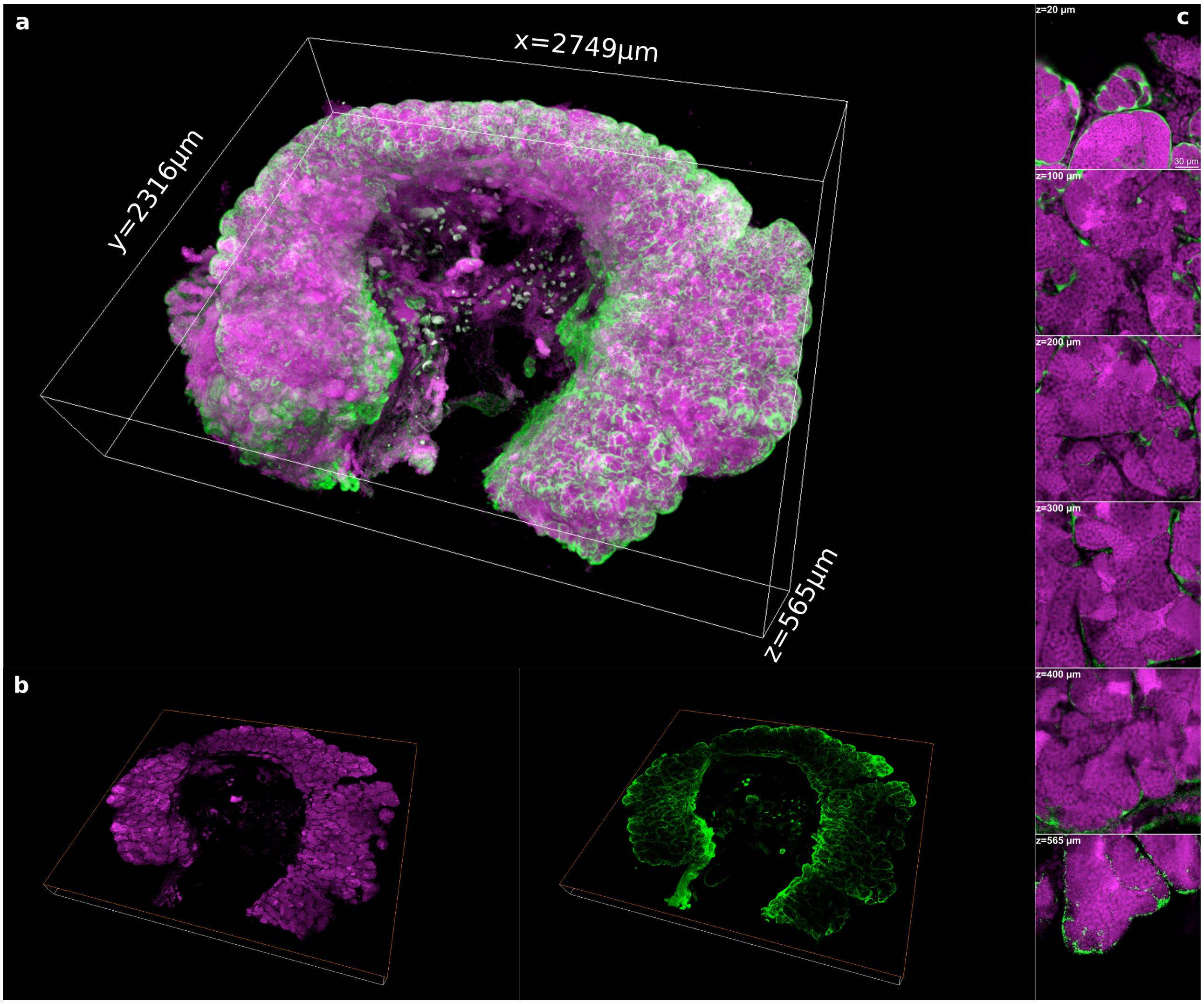

Supplementary Fig. S5: 3D reconstruction of a whole portion of a testis cleared by the PACT method.

(a) 3D rendering of a whole portion of a testis dissected from the transgenic line *Tg(gsdf:GFP)* and cleared by the PACT method. **(b)** 2D optical section of the whole testis at 350 µm in depth. **(c)** Magnified view on 2D optical sections at 20 µm, 100 µm, 200 µm, 300 µm, 400 µm and 565 µm in depth respectively. We acquired a total volume of 2.749 mm x 2.316 mm x 0.565 mm. The imaging of the whole testis took 7.5 h in our conditions and generated 28 GB of data. Images were acquired in 12 bits at a scanning speed of 600 Hz and a resolution of 512 x 512 pixels with two lines average. Voxel size: 0.865 µm x 0.865 µm x 1 µm. Nuclei are in magenta and Sertoli cells in green. Scale bar in (c) : 30 µm.

Supplementary Video S6: 3D reconstruction of a whole portion of a testis cleared by the PACT method.

The movie shows the 3D rendering of a testis dissected from the zebrafish transgenic line *Tg(gsdf:GFP)*. Volume size: (2.316 mm x 2.749 mm x 0.565 mm). Nuclei are in magenta and Sertoli GFP cells in green.

Supplementary Video S7: z-stacks of a subvolume of testis cleared by PACT method.

The movie shows the 3D rendering of a testis dissected from the zebrafish transgenic line *Tg(gsdf:GFP)*. Nuclei are in magenta and Sertoli GFP cells in green. A series of grayscale 2D-optical slices showing GFP in Sertoli cells (z-step: 1 μm). A series of grayscale 2D optical slices showing nuclear staining by propidium iodide (z-step: 1 μm).

Supplementary Video S8: 3D reconstruction of two nearby niches located in adjacent seminiferous tubules and displaying six nuclei each.

Supplementary Video S9: 3D reconstruction of a niche displaying two nuclei of undifferentiated spermatogonia.
